# EEG Signal Multichannel Frequency-Domain Ratio Indices for Drowsiness Detection Based on Multicriteria Optimization

**DOI:** 10.3390/s21206932

**Published:** 2021-10-19

**Authors:** Igor Stancin, Nikolina Frid, Mario Cifrek, Alan Jovic

**Affiliations:** Faculty of Electrical Engineering and Computing, University of Zagreb, Unska 3, 10000 Zagreb, Croatia; igor.stancin@fer.hr (I.S.); nikolina.frid@fer.hr (N.F.); mario.cifrek@fer.hr (M.C.)

**Keywords:** drowsiness detection, EEG, frequency-domain features, multicriteria optimization, machine learning

## Abstract

Drowsiness is a risk to human lives in many occupations and activities where full awareness is essential for the safe operation of systems and vehicles, such as driving a car or flying an airplane. Although it is one of the main causes of many road accidents, there is still no reliable definition of drowsiness or a system to reliably detect it. Many researchers have observed correlations between frequency-domain features of the EEG signal and drowsiness, such as an increase in the spectral power of the theta band or a decrease in the spectral power of the beta band. In addition, features calculated as ratio indices between these frequency-domain features show further improvements in detecting drowsiness compared to frequency-domain features alone. This work aims to develop novel multichannel ratio indices that take advantage of the diversity of frequency-domain features from different brain regions. In contrast to the state-of-the-art, we use an evolutionary metaheuristic algorithm to find the nearly optimal set of features and channels from which the indices are calculated. Our results show that drowsiness is best described by the powers in delta and alpha bands. Compared to seven existing single-channel ratio indices, our two novel six-channel indices show improvements in (1) statistically significant differences observed between wakefulness and drowsiness segments, (2) precision of drowsiness detection and classification accuracy of the XGBoost algorithm and (3) model performance by saving time and memory during classification. Our work suggests that a more precise definition of drowsiness is needed, and that accurate early detection of drowsiness should be based on multichannel frequency-domain features.

## 1. Introduction

Drowsiness is the intermediate state between wakefulness and sleep [1]. Terms such as sleepiness or tiredness are used synonymously with drowsiness in related studies [2,3,4]. Although it is intuitively clear what drowsiness is, it is not so easy to determine exactly whether a person is in a drowsy state or not. The reason for this is the unclear definition of drowsiness. Some researchers define drowsiness as stage 1 sleep (S1) [5,6,7,8,9], which is also known as non-rapid eye movement 1 (NREM 1) sleep. Da Silveira et al. [10] used S1 sleep stage data in their research of drowsiness. Johns [11] claims that the S1 sleep stage is equivalent to microsleep (episodes of psychomotor insensitivity due to sleep-related wakefulness loss [12]), while drowsiness is stated to occur before S1 sleep, but it is not stated when it begins and what characterizes it. Researchers who do not use any of the aforementioned definitions of drowsiness typically use a subjective assessment of drowsiness, e.g., the Karolinska sleepiness scale [13]. In this paper, the term drowsiness is used as a synonym for the S1 sleep stage.

In a drowsy state, people are not able to function at the level required to safely perform an activity [14], due to the progressive loss of cortical processing efficiency [15]. Drowsiness is, therefore, a significant risk factor for human lives in many occupations, e.g., for air traffic controllers, pilots and regular car drivers [16]. According to the reports from NASA [17] and the National Transportation Safety Board [18], one of the main factors in road and air accidents is drowsiness. Gonçalves et al. [19] conducted a study across 19 European countries and concluded that in the last two years, 17% of drivers fell asleep while driving, while 7% of them had an accident due to drowsiness. The high frequency and prevalence of drowsiness-related accidents speak in favor of the development of early drowsiness detection systems, which is the subject of this paper.

Many researchers are trying to solve the problem of early detection of drowsiness in drivers. Balandong et al. [20], in their recent review, divided the techniques for detecting driver drowsiness into six categories: (1) subjective measures, (2) vehicle-based systems, (3) driver’s behavior-based systems, (4) mathematical models of sleep–wake dynamics, (5) human physiological signal-based systems and (6) hybrids of one or more of these techniques. Currently, the most common techniques used in practice are vehicle-based systems [5], but these systems are mostly unreliable and depend largely on the driver’s motivation to drive as well as possible [20].

Physiological signals are the promising alternative for reliable drowsiness detection [21]. The main problem with this approach is that these systems are often not easy to use and are intrusive to drivers [20]. Nevertheless, many researchers are working on small, automated and wearable devices [21,22,23,24], or on steering wheel devices [25,26] in order to overcome these obstacles. Techniques for detecting drowsiness based on physiological signals can be further subdivided according to the type of signal used, such as electroencephalogram (EEG) [27], electrooculogram (EOG) [28] or electrocardiogram (ECG) [29].

The most studied and applied physiological signal to detect drowsiness is the EEG. In this paper, frequency-domain features of the EEG signal are analyzed and two novel multichannel ratio indices for the detection of drowsiness are proposed. Besides the frequency-domain features, there are also other types of features: (1) nonlinear features [30], (2) spatiotemporal (functional connectivity) features [31] and (3) entropies [32]. These three groups of features have a lower frequency of use compared to the frequency-domain features, so in this paper, we focus only on frequency-domain features. Based on the recent review [33] of EEG-based drowsiness detection systems, 61% of the included papers used frequency-domain features, 38% used entropies, 10% used nonlinear features and 10% used spatiotemporal features (some papers used multiple groups of features, so the sum of the percentages is greater than 100%). This shows the difference in the use of drowsiness detection systems, and the difference is even greater in the general field of neurophysiological scientific papers. Although the three feature groups mentioned above are used less frequently, there are still a certain number of papers that include them, especially entropies.

Frequency-domain features estimate the power spectral density in a given frequency band. The bands typically used in the analysis of EEG signals are delta (δ, 0.5–4 Hz), theta (θ, 4–8 Hz), alpha (α, 8–12 Hz), beta (β, 12–30 Hz) and gamma (γ, >30 Hz). An increase in theta activity [34] and an increase in alpha activity [35] indicate drowsiness. An increase in the beta activity, however, is a sign of wakefulness and alertness [36]. There are several widely used frequency-domain ratio indices for detecting drowsiness. Eoh et al. [36] proposed the θ/α and β/α ratio indices, Jap et al. [37] proposed the (θ + α)/β, θ/β and (θ + α)/(α + β) ratio indices and da Silveira et al. [10] proposed the γ/δ and (γ + β)/(δ + α) ratio indices. These ratio indices provide improvement in the detection of drowsiness compared to the frequency-domain features alone and are shown to correlate with drowsiness.

All these frequency-domain features and ratio indices are calculated from a single EEG channel, i.e., from a single brain region. In recent research, Wang et al. [38] showed that the significance of a decrease in delta and an increase in (θ + α)/β indices depends on the brain region. This significant diversity of the correlation of features with drowsiness in different brain regions is the motivation for this research. Since all currently used frequency-domain features and ratio indices are based on a single channel (single brain region), this work aims to use the best distinguishing features of each brain region for the detection of drowsiness and to combine them into a single multichannel ratio index feature.

In our work, we use a computational method based on multicriteria optimization to extract the multichannel EEG-based frequency-domain ratio index features. This method allows us to discover new multichannel ratio indices that show improvements in the detection of drowsiness compared to single-channel ratio indices. Finally, with the use of machine learning models, we prove that multichannel indices detect drowsiness with higher accuracy, higher precision, reduced memory and faster computation compared to single-channel features.

In the Materials and Methods Section, we show the methodology of our work, including a description of the dataset, preprocessing and feature extraction methods used. Novel multichannel ratio indices and the multi-objective optimization method are also described there. In the Results Section, we present the results of our work, including statistical analysis, drowsiness prediction and computational properties of the proposed indices. In the Discussion Section, we discuss in more detail the topics covered in this paper. Finally, in the last section, we conclude the paper.

## 2. Materials and Methods

### 2.1. Dataset, Preprocessing and Feature Extraction

The data used in this paper were obtained from the PhysioNet portal [39], in particular from the 2018 PhysioNet computing in cardiology challenge [40]. The original dataset contains data records from 1985 subjects, and each recording includes a six-channel EEG, an electrooculogram, an electromyogram, a respiration signal from the abdomen and chest, airflow and oxygen saturation signals and a single-channel electrocardiogram during the all-night sleep. The records were divided into training and test sets of equal size. The sleep stages [41] of all subjects were annotated by clinical staff based on the American Academy of Sleep Medicine (AASM) manual for the scoring of sleep [42]. There are six types of annotations for different stages: wakefulness (W), stage 1 (S1), stage 2 (S2), stage 3 (S3), rapid eye movement (REM) and undefined.

In this research, we wanted to use a training set (992 subjects) to detect drowsiness. The officially provided way of acquiring the data is through torrent download, but we managed to download only 393 subjects completely, due to a lack of seeders. Of these 393 subjects, EEG signal recordings from 28 subjects were selected, based on the condition that each recording had at least 300 s of the W stage and, immediately after that, at least 300 s of the S1 stage. From each recording, a fragment of 600 s (300 s of W stage and 300 s of S1 stage) was used for analysis. In the original dataset, each EEG signal recording consists of six channels (*F*3, *F*4, *C*3, *C*4, *O*1 and *O*2, based on the International 10/20 System), with a sampling frequency of 200 Hz. Table 1 shows the identification numbers of all the selected subjects. The subjects were divided into two groups, one group used for training of the model (16 subjects) and the other one for the test of the obtained models (12 subjects). The training set was used to obtain novel ratio indices (with the method described below) and the test set was used to check these novel indices on the unseen data.

Before feature extraction, the EEG signal must be filtered. For this purpose, the DC component was removed from the signal and the signal was filtered with a Butterworth filter to remove high-frequency artifacts and low-frequency drifts. We used the sixth-order Butterworth filter, the low-cut frequency of 1 Hz and the high-cut frequency of 40 Hz. In the selected fragments of the recordings, there was an insignificant number of eye-related artifacts, so we decided not to use the independent component analysis for their removal in order to prevent potential information loss due to component removal.

The signals were divided into epochs to calculate features. The epochs were five seconds long with a 50% overlap between them. Frequency-domain features are often used in EEG signal analysis. These features were extracted from the power spectral density (PSD) of the signal. To obtain the PSD of the signal, Welch’s method [43] was used. Welch’s method is used more often than Fast Fourier transform in the field of EEG signal analysis since it produces PSD with lower variance. The standard frequency-domain features were calculated, i.e., delta (δ, 0.5–4 Hz), theta (θ, 4–8 Hz), alpha (α, 8–12 Hz) and beta (β, 12–30 Hz) bands. We also calculated the less frequently used frequency-domain features, i.e., gamma (γ, >30 Hz), sigma (σ, 12–14 Hz), low alpha (α1, 8–10 Hz) and high alpha (α2, 10–12 Hz) bands [44].

### 2.2. Novel Multichannel Ratio Indices

**Figure 1:**
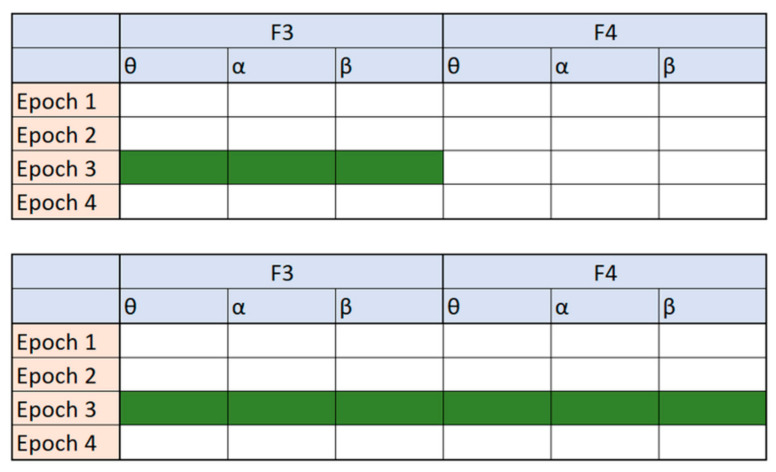
A visualization of tables with features. The green color represents the possibilities for creating a ratio index, the first table (**top**) are the possibilities reported in the related work to create a single-channel ratio index, while the second table (**bottom**) are the possibilities explored in our novel multichannel approach.

Ratios between frequency-domain features have often been used as new features in different areas of EEG signal analysis [10,36]. All these features have a simple mathematical formulation but often lead to an improvement in detection and reduction of dimensionality for drowsiness. Moreover, they are calculated based on a single channel only. The idea behind the novel indices we present in this work is to design the feature formulation in such a way that frequency-domain features from different channels can be combined. Figure 1 illustrates the difference between these two approaches. For simplicity of visualization, only four epochs, two channels (*F*3 and *F*4) and three features per channel are shown in Figure 1.

We define a new index, *I*, for each epoch, *e*, which is calculated as a ratio of the feature values, *F*(*e*), for all six channels in the epoch, *e*. In both the nominator and denominator, the feature value of each channel, *j*, is multiplied with a dedicated coefficient, *C_ij_* or *K_ij_* respectively, as indicated in the Equation (1):

(1) $$I\left(e\right)=\frac{\sum_{i=features}\sum_{j=channels}C_{ij}F_{ij}\left(e\right)}{\sum_{i=features}\sum_{j=channels}K_{ij}F_{ij}\left(e\right)}$$

The purpose of the coefficients is to reduce or even eliminate the influence of certain channels of frequency-domain features, by setting the value in the range [0, 1〉, or increase the influence of certain channels of the frequency-domain features by setting the corresponding coefficient to a value in the range [1,∞〉. There are 48 (6 channels and 8 features per channel) *C* coefficients and 48 *K* coefficients.

**Figure 2:**
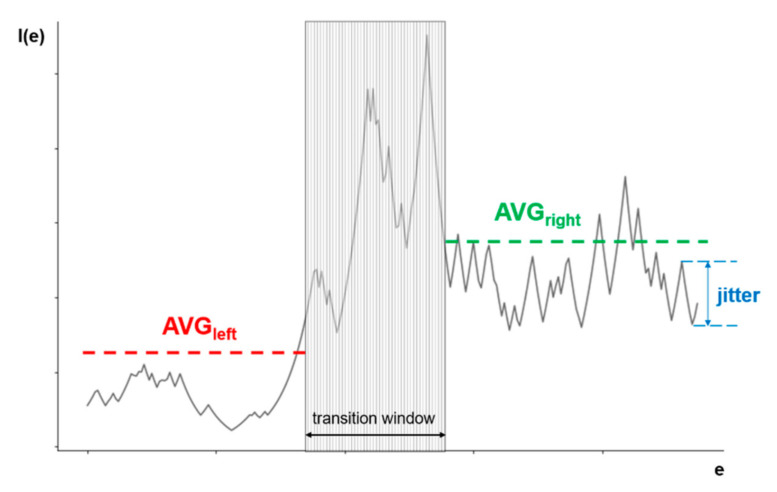
An illustration of all the elements needed for an evaluation of solutions of the multi-objective optimization in drowsiness detection.

The ideal output of *I*(*e*) should look like a step function (or an inverse step function), which would indicate a clear difference between the two stages: W and S1. Figure 2 illustrates the main features of the output. The output can be divided into two parts: the left one corresponds to stage W and the right one to S1. While the output in each part should be as smooth as possible, i.e., with minimal oscillations, it is expected that there will be a transition period between the phases, which may have significant oscillations. This transition period would ideally be the step function, but in realistic settings, it is expected that the transition between phases of brain activity will probably last several epochs and would not be considered as either stage W or S1.

In order to determine the appropriate value of the coefficients that would provide the output as close as possible to the ideal, at least two criteria must be taken into account: the absolute difference between the mean values left and right of the transition window and the quantification of the oscillations in each part. This can be defined as a multi-objective optimization problem that we want to solve using a metaheuristic multi-objective evolutionary optimization method, as described in the next section. To the best of our knowledge, this state transition problem has never been approached with evolutionary computation.

### 2.3. Multi-Objective Optimization

The optimization of a step function that is representative of the problem of flat surfaces is generally a challenge for any optimization algorithm because it does not provide information about which direction is favorable and an algorithm can get stuck on one of the flat plateaus [45]. To overcome this challenge, instead of optimizing the function according to one criterion, we define two objectives that we optimize simultaneously: (1) to maximize the absolute difference between the mean value of *I*(*e*) output for the W and S1 stages, and (2) to minimize the oscillations of the output value around the mean value in each stage. According to Figure 2, the left part of the *I*(*e*) output occurring before the transition phase corresponds to the W stage, and the right part, occurring after the transition phase, corresponds to the S1 stage. Since optimization problems are usually expressed as minimization problems, where the first objective function, *O*1, is defined as the inverse absolute difference between the mean value of *I*(*e*) of the left part (*avg_left_*) and the right part (*avg_right_*), Equation (2) is established:

(2) $$O1=\frac{1}{\left|avg_{right}-avg_{left}\right|}$$

The second objective function, *O*2, expresses the oscillations in the function and is defined as the number of times the difference between the output values of *I*(*e*) for two adjacent epochs was greater than a given limit. The exact value of this limit will be discussed later in this section as it is closely related to the specifics of the optimization method used. The main goal of the objective function *O*2 is to minimize the influence of the biggest flaw in the way that the objective *O*1 is calculated, i.e., to use the averaging function. For example, if a possible solution is a completely straight line, except for a large negative spike in the left part and a large positive spike in the right part, based only on the objective function *O*1, this would be a good solution, while the objective function *O*2 would penalize this solution.

As mentioned above, the transition between two stages will probably take several epochs and show significant oscillations of the function output values. According to the annotation made by clinical personnel, the transition phase should be approximately in the middle of the *I*(*e*) output, but it cannot be determined exactly how long it will last. In our work, which is based on expert knowledge of human behavior in the case of drowsiness, we assume that it lasts about one minute, which corresponds to about 30 epochs. Within the transition window, neither one of the two objective functions is calculated, since it is assumed to belong neither to the W nor to the S1 stages. We also allow it to move around the center, shifting left and right, due to a possible error of the human observer who marked the data.

The multi-objective optimization problem can now be expressed as min{*O*1,*O*2}, where *O*1 and *O*2 are the conflicting objective functions, as defined above. The evolutionary metaheuristic algorithm NSGA-II [46] was applied to solve this multi-objective optimization problem. The genetic algorithms (GAs) are normally used to solve complex optimization and search problems [47]. NSGA-II is one of the most popular evolutionary multicriteria optimization methods due to its versatility and ability to easily adapt to different types of optimization problems. The strong points of this MO algorithm are: (1) the fast non-dominated sorting ranking selection method used to emphasize Pareto-optimal solutions, (2) maintaining the population diversity by using the crowding distance and (3) the elitism approach, which ensures the preservation of best candidates through generations without the setting of any new parameters other than the normal genetic algorithm parameters, such as population size, termination parameter, crossover and mutation probabilities. Additionally, it was often used for the elimination of EEG channels with the similar purpose as in our case-dimensionality reduction [48]. This paper uses the implementation of NSGA-II provided by the MOEA framework [49] and is based on the guidelines defined in [46,50].

**Figure 3:**
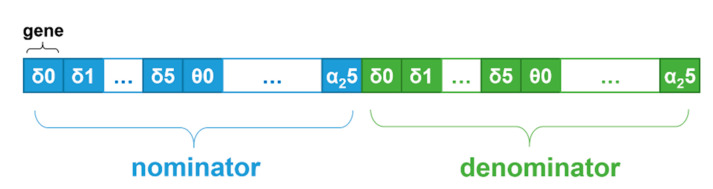
An illustration of a chromosome structure in the proposed optimization problem solution.

NSGA-II was used with the following configuration. The chromosome was divided into two parts: in the first part, genes represented the nominator coefficient values (*C_ij_*), and in the second part, genes represented the denominator coefficient values (*K_ij_*). In each part, the genes were grouped by frequency-domain features and channels, as illustrated in Figure 3. The genes were encoded as real values in the range [0.0, 10.0], and standard NSGA-II crossover and mutation operators were used to support operation on real values.

Each solution is evaluated based on the values of objectives *O*1 and *O*2, as described in the pseudocode in Algorithm 1. First, the chromosome is decoded (line 1). Then, for each test fragment, two values are calculated: (1) the inverse absolute difference (IAD) between the mean index value, *I*(*e*), of the left part and the right part, represented by the invAbsDiff variable in the pseudocode, and (2) the oscillations in the function, represented by the oscillation variable in the pseudocode (lines 3–5). Finally, the value of each objective *O*1 for the given solution is defined as the average value of invAbsDiff for all test fragments, and the value of objective *O*2 is defined as the average value of oscillation for all test fragments (lines 7–8).
**Algorithm 1.** Evaluation.1: decode chromosome to get coefficient values2: **for** each fragment **do**3:   indexVals[[] = calculate index value for each epoch4:   invAbsDiff += IADCalc(indexVals[[], windowStart)5:   oscillation += OscillationCalc(indexVals[[], windowStart, winSize)6: **end for**7: objective1 = invAbsDiff/number_of_fragments8: objective2 = oscillation/number_of_fragments

The algorithm for the IAD calculation is provided in the pseudocode in Algorithm 2. The calculation of the IAD for each fragment was slightly modified compared to Equation (1) to allow a faster convergence of the search algorithm. The transition phase was not in the same position in each fragment but allowed to move more loosely away from the center because the annotation in the original dataset was performed manually and there was a possibility of human error in case the observer would register a transition from W to S1 a little too early or too late. The algorithm allows the transition phase to begin no earlier than 30 epochs from the fragment start, and end no later than 60 epochs before the fragment end (line 2). The algorithm assumes the transition phase by looking for a window of 30 epochs which has the maximum difference of index, *I*(*e*), values between the left and the right part (lines 9–13).

The gradation of the absolute difference between the mean value of the left and the right parts is also introduced (lines 19–22) to allow easier and faster convergence of the algorithm. The optimization of the objective *O*1 can be considered as an optimization problem with soft constraints that are related to how much *O*1 deviates from the optimal value. However, it is quite difficult to determine the optimal value precisely a priori. As indicated in [51,52], constraints are often treated with penalties in optimization techniques. The basic idea is to transform a constrained optimization problem into an unconstrained one by introducing a penalty into the original objective function to penalize violations of constraints. According to a comprehensive overview in [51], the penalty should be based on the degree of constraint violation of an individual. In [53], it is also recommended that instead of having just one fixed penalty coefficient, the penalty coefficient should increase when higher levels of constraint violation are reached. The greatest challenge, however, is to determine the exact penalty values. If the penalty is too high or too low, evolutionary algorithms spend either too much or too little time exploring the infeasible region, so it is necessary to find the right trade-off between the objective function and the penalty function so that the search moves towards the optimum in the feasible space. As the authors have shown in [54], the choice of penalty boundaries is problem-dependent and difficult to generalize. Since we cannot strictly determine the optimal value of *O*1 in our case, we have chosen several thresholds for the absolute difference value, with the penalty increasing by a factor of 10 for each new threshold. The exact thresholds were selected based on the experience gained from the first few trial runs of the algorithm. Based on the observations from the trial runs, a third modification was also introduced: the difference is calculated with a relative, instead of absolute, value of *I*(*e*). The relative value of *I*(*e*) is calculated by using the lowest *I*(*e*) value as a reference point, instead of zero, i.e., the zero is “moved”, as shown in code lines 16–18 in Algorithm 2.
**Algorithm 2.** IAD Calculation.1: **function**
IADCalc(indexVals[[], windowStart)2:  **for** j between 30 and (indexVals.size-60) **do**3:   maxAbsDiff = 04:   left = 05:   right = 06:   avgLeft = average value of all Index values before j7:   avgRight = average value of all Index values after j+308:   diff = ABS(avgRight–avgLeft)9:   **if** diff ≥ maxAbsDiff **then**10:    maxAbsDiff 0 diff11:    left = avgLeft12:   right = avgRight13:    windowStart = j14:   **end if**15:  **end for**16:  lowestVal = getLowestVal(indexVals)17:  movedZero = lowestVal–0.01*lowestVal18:  absDiff = ABS(right–left)/MIN(left–movedZero, right–movedZero)19:  **if** absDiff ≥ 5.0 **then** invAbsDiff = 1/absDiff20:  **else if** absDiff ≥ 1.0 **then** invAbsDiff = 10/absDiff21:  **else if** absDiff ≥ 0.5 **then** invAbsDiff = 100/absDiff22:  **else** invAbsDiff = 100023:  **end if**24:  **return** invAbsDiff25: **end function**

The pseudocode for calculating the oscillations in the function as the second objective, *O*2, is provided in Algorithm 3. Again, the optimization of the oscillations can be considered a constrained optimization problem, so that, in the same way as in the case of the IAD calculation discussed previously, a gradation of the difference between the output values of *I*(*e*) for two adjacent epochs is used to penalize the larger differences more severely (lines 7–10 and 15–18). The exact thresholds were chosen based on the experience gained from the first few trial runs of the algorithm. In order to make the algorithm converge more easily and quickly, the concept of “moved zero” was used again (lines 2, 3, 6 and 14).
**Algorithm 3.** Oscillation Calculation.1: **function**
OscillationCalc(indexVals[[], windowStart, winSize)2:  lowestVal = getLowestVal(indexVals)3:   movedZero = lowestVal–0.01*lowestVal4:   oscillation = 05:   **for** i between 1 and windowStart–1 **do**6:    absDiff = ABS((indexVals[i]-indexVals[i-1])/(indexVals[i-1] -movedZero))7:    **if** absDiff ≥ 5.0 **then** oscillation += 10008:    **else if** absDiff ≥ 1.0 **then** oscillation += 1009:    **else if** absDiff ≥ 0.5 **then** oscillation += 1010:    **else if** absDiff ≥ 0.25 **then** oscillation += 111:    **end if**12:   **end for**13:   **for** i between windowStart+winSize and indexVals.size()-1 **do**14:    absDiff = ABS((indexVals[i]-indexVals[i-1])/(indexVals[i-1] -movedZero))15:    **if** absDiff ≥ 5.0 **then** oscillation += 100016:     **else if** absDiff ≥ 1.0 **then** oscillation += 10017:     **else if** absDiff ≥ 0.5 **then** oscillation += 1018:    **else if** absDiff ≥ 0.25 **then** oscillation += 119:    **end if**20:   **end for**21:   **return** oscillation22: **end function**


Finally, to further minimize the oscillations, and help the search algorithm converge more quickly, the maximum change in the *I*(*e*) value between two adjacent epochs is set to 10% of the first of the two epochs. The mathematical formulation of this limit is provided in Equation (3):

(3) $$Index\left(e\right)=\left\{\begin{array}{c} 1.1*I\left(e-1\right),\;if\;I\left(e\right)>1.1*I\left(e-1\right)\\ 0.9*I\left(e-1\right),\;if\;I\left(e\right)<0.9*I\left(e-1\right)\\ I\left(e\right),\;else\; \end{array}\right.$$

## 3. Results

The optimization algorithm was executed over 107 generations, using 100 randomly selected chromosomes as a starting point. Ideally, the optimization algorithm would have many *C* and *K* coefficients equal to zero and only a few non-zero coefficients in order to obtain a simple and easily understandable mathematical formulation of a novel multichannel ratio index. Unfortunately, even the best solutions of the optimization algorithm had only up to 20 *C* and *K* coefficients equal to zero. Although such a novel multichannel ratio index showed good behavior in detecting drowsiness, it is impractical to use a formula with 76 coefficients. We consider anything above 15 coefficients to be impractical.

In order to reduce the number of coefficients and to simplify the formulation of the novel multichannel ratio index, some coefficients were manually set to zero. In order to decide which coefficients have the least influence on the final solution, we counted how often a large value of the coefficient is fixed to a certain frequency-domain feature. By analyzing the coefficients of all solutions in the final population of the optimization algorithm, we concluded that the most frequently selected features were δ, α, α1 and α2. After manually fixing the coefficients of all other frequency-domain features to zero, the search range for the optimization algorithm was reduced to half.

Although 48 *C* and *K* coefficients remained in the solution at that time, the algorithm provided equally good results in terms of drowsiness detection, but with a much simpler mathematical formulation. In addition to the 48 coefficients that were manually set to zero, the algorithm often set many more coefficients to zero. A decision on the best solution in the final population was made based on the *O*1 and *O*2 values of the optimization algorithm in combination with the number of coefficients set to zero after using the floor operator on the coefficients. The floor operator was used to simplify the equation by removing the decimal numbers. Preferred solutions are those with a higher number of coefficients set to zero. Our choice was the solution with 13 non-zero coefficients, as shown in Equation (4):

(4) $$I1\left(e\right)=\frac{\alpha_{F3}+4\alpha_{O2}+9\alpha 1_{F3}+3\alpha 1_{C3}+9\alpha 1_{C4}+\alpha 1_{O2}+4\alpha 2_{O1}+8\alpha 2_{O2}}{\delta_{F3}+3\delta_{F4}+3\delta_{C3}+2\delta_{C4}+9\delta_{O2}}$$

All *C* and *K* coefficients were rounded to a lower value (floor operator). Here, *e* represents the current epoch and all the features on the right side were from that same epoch.

The goal of the second condition of the optimization algorithm was to minimize the oscillations of the *I*(*e*) function. The results were much better with this condition than without it, but the resulting function still oscillated strongly. In order to additionally minimize the oscillations, a limitation was performed. The maximum change between any two adjacent samples was set to 10% of the value of the first sample. Equation (5) shows the mathematical formulation of this limitation of the maximum change:

(5) $$Index1\left(e\right)=\left\{\begin{array}{c} 1.1*I1\left(e-1\right),\;if\;I1\left(e\right)>1.1*I1\left(e-1\right)\\ 0.9*I1\left(e-1\right),\;if\;I1\left(e\right)<0.9*I1\left(e-1\right)\\ I1\left(e\right),\;else\; \end{array}\right.$$

where *I*1(*e*) is defined by Equation (4) and *e* is the current epoch. Limiting the maximum change of adjacent samples further improves the detection model, and therefore Equation (5) presents the first novel multichannel ratio index.

We have tried to further simplify the formulation of the multichannel ratio index. This time, brute force search for the best solution was applied with the following constraints: (1) encoding of all *C* and *K* coefficients was set to integer values of zero or one for the sake of simplicity, and (2) a maximum of five addends in the equation was allowed. With these constraints, we obtained Equation (6):

(6) $$I2\left(e\right)=\frac{\delta_{F3}+\delta_{F4}+\delta_{O2}}{\alpha_{C3}+\alpha 2_{O2}}$$

Again, similar to the first index, the maximum change was limited, so that the final equation for the second ratio index was obtained as:

(7) $$Index2\left(e\right)=\left\{\begin{array}{c} 1.1*I2\left(e-1\right),\;if\;I2\left(e\right)>1.1*I2\left(e-1\right)\\ 0.9*I2\left(e-1\right),\;if\;I2\left(e\right)<0.9*I2\left(e-1\right)\\ I2\left(e\right),\;else\; \end{array}\right.$$

where *I*2(*e*) is defined by Equation (6) and *e* is the current epoch. After obtaining the two novel indices, they were normalized to the range [0, 1] for each subject to eliminate interindividual differences between the subjects.

**Figure 4:**
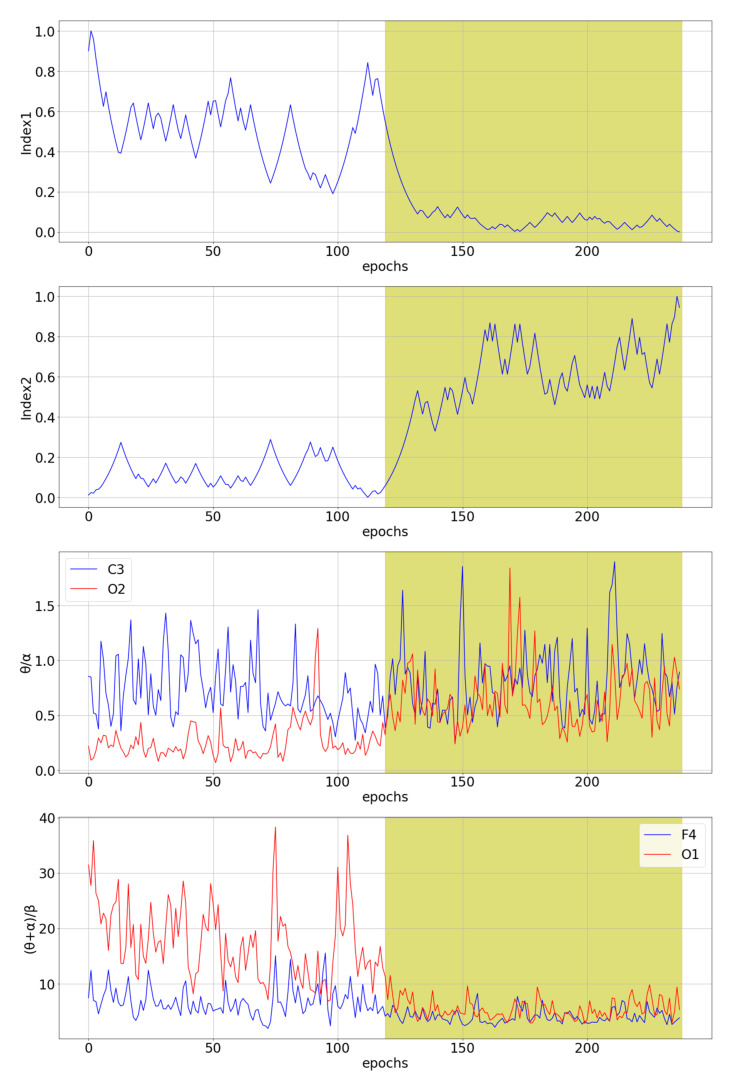
The comparison of the two novel multichannel indices with the best and the worst channel for θ/α and (θ + α)/β single-channel indices for subject tr08-0111. The white part of the diagram represents an awake state, while the yellow part of the diagram represents stage 1 of sleep, i.e., a drowsiness state.

The two novel multichannel ratio indices defined by Equations (5) and (7) were compared with the seven existing indices θ/α and β/α [36], (θ + α)/β, θ/β and (θ + α)/(α + β) [37], and γ/δ and (γ + β)/(δ + α) [10]. The indices γ/δ and (γ + β)/(δ + α) were calculated based on the wavelet transform, i.e., in the same way as in the original paper. Figure 4 shows a comparison of our novel indices with the best and the worst channel for θ/α and (θ + α)/β single-channel indices for subject tr08-0111. These two single-channel indices were selected because they are the best predictors of drowsiness for a given subject among all single-channel indices.

### 3.1. Statistical Analysis

The Wilcoxon signed-rank test [55] was used to analyze the statistical differences between the awake state and the S1 state. This test was chosen because it refers to data that do not necessarily follow the normal distribution. Table 2 shows *p*-values for each subject in the training set and each index. The significance level α_0_ = 0.01 was used together with the Bonferroni correction [55] to reduce the probability of false-positive results, as the test was repeated 144 times (16 subjects and 9 indices), giving us the final α_p_ = 6.9 × 10^−5^. For the existing indices, the *p*-value was calculated for each channel, but only the *p*-values of the best channel (the lowest average of *p*-value for all subjects) are shown in Table 2.

The two novel indices show *p*-values lower than α_p_ for most subjects. From this, we can conclude that, for Index1, 14 of 16 subjects show two different distributions for the W stage and the S1 stage, while 13 of 16 subjects show significantly different distributions of the W stage and the S1 stage for Index2. There are only two existing indices where the *p*-value is lower than αp in more than ten cases. These are θ/β and (θ + α)/(α + β), both by Jap et al. [37].

Table 3 shows *p*-values for each subject in the test set and each index. Again, the two novel indices, together with the (γ + β)/(δ + α) [10] index, show *p*-values lower than α_p_ for most subjects.

### 3.2. Drowsiness Prediction Analysis

An additional comparison of ratio indices was performed by analyzing the drowsiness detection accuracy and precision, as obtained with the XGBoost algorithm [56]. Default parameters were applied: learning rate eta equal to 0.3, gamma equal to 0 and a maximum depth of a tree equal to 6. For a detailed comparison of the indices, classification accuracy and precision were calculated for each subject. Namely, each subject has 238 epochs of the measured signal, with the first half representing the W state and the second half the S1 state. The algorithm classified the subject’s state for each epoch (238 classifications per subject), and the accuracy for each subject was calculated based on these classifications. The leave-one-subject-out cross-validation method was applied on the training set, i.e., the algorithm was trained on the data of 15 subjects and tested on the subject excluded from the training set, and this was repeated 16 times to evaluate drowsiness detection on each subject from the training set. Table 4 shows the classification accuracy achieved on the training set.

Index1 has the highest average accuracy and the highest classification accuracy for 3 of 16 subjects. Index2 has the second-highest average accuracy and the highest classification accuracy for 4 of 16 subjects, which is the most of all indices. θ/α [36] and (θ + α)/(α + β) [37] are the only other indices with an average classification accuracy above 0.58, while θ/α [36] and θ/β [37] are the only other indices with the highest accuracy for 3 of 16 subjects. The β/α [36] index has the lowest average classification accuracy on the training set (0.5515).

Table 5 shows the classification accuracy on the test set. Index1 has the highest average accuracy and the highest classification accuracy for 3 of 12 subjects. Index2 has the third-highest average accuracy and the highest classification accuracy for 4 of 12 subjects, which is the most of all indices. The only other index with comparable accuracy is θ/α [36], with the second-highest average accuracy. All other indices have at least 2.5% lower accuracy than the two novel indices.

Table 6 shows the degree of precision of drowsiness detection on the training set. Index2 has the highest average precision of drowsiness detection and the highest precision of drowsiness detection for five subjects, which is the highest of all indices. Index1 has the second-best average precision of drowsiness detection. (θ + α)/(α + β) [37] and γ/δ [10] have a precision of drowsiness detection comparable to Index1 and Index2, while all other ratio indices have lower precision.

Table 7 shows the degree of precision of drowsiness detection achieved on the test set. Index1 has the highest average precision. θ/α [36] and γ/δ [10] have 1% lower precision than Index1, while all other indices have at least 4% lower precision. Index2 has the second-highest average precision.

### 3.3. Computational Analysis

With regard to the classification and the use of machine learning algorithms, an advantage of using the novel multichannel indices compared to the existing single-channel indices is also the saving of memory and time, due to the reduction of dimensionality. The accuracies of Index1 and Index2 from Table 4 were achieved with the model constructed from the single feature only, while all other indices had six features since the dataset contains six EEG channels. For this reason, storing the novel indices consumes six times less memory. The time consumption was measured as an average of 100 executions. The measured time included classifier initialization, classifier training, classifications on the test subject and calculation of classification accuracy. Table 8 shows the results of time consumption measurements. The use of the novel multichannel indices saves about 30% of time compared to all other traditionally used single-channel ratio indices.

## 4. Discussion

The main idea of our research was to combine frequency-domain features from different brain regions into a multichannel ratio index to improve frequency-domain features for the detection of drowsiness and to gain new insights into drowsiness. The results in Table 2, Table 3, Table 4, Table 5, Table 6, Table 7 and Table 8 suggest that two novel multichannel ratio indices improve the detection of drowsiness based on the frequency-domain features and reduce the time required for detection.

We must note that the main idea of this research was not to create the best possible model for drowsiness detection but only to bring improvement into frequency-domain features that are often used for drowsiness detection. Our focus was on developing the method for obtaining these novel indices, which is explained in Section 2.3 “Multi-Objective Optimization”. In order to confirm that our conclusions also hold for other classifiers besides XGBoost, Table 9 shows the average accuracy on the test set obtained with Naïve Bayes, k nearest neighbors, logistic regression, decision tree, random forest and support vector machine classifiers (using the scikit-learn library at default settings). The average accuracies of two novel indices vary from 56% to 65% among the algorithms. All the algorithms show that our novel multichannel indices are better than existing single-channel indices.

Our results were compared with the seven existing single-channel ratio indices that are currently state-of-the-art frequency-domain features. The newest one was introduced in 2016 [10], but all of these single-channel ratio indices are often used in the more recent drowsiness detection papers [57,58,59].

The authors in the aforementioned research report 92% accuracy as the best-obtained accuracy [57]. This accuracy was obtained based on the epoch-level validation. Epoch-level validation is a cross-validation procedure on the epoch level, which means that there is a very high probability that all subjects will have epochs in the training set and in the test set at the same time. On the other hand, subject-level validation is validation where it is ensured that subjects in the test set are not contained in the training set. An example of a subject-level validation is the leave-one-subject-out cross-validation that we used in this research. The only proper way for model validation is subject-level validation, as it represents the real-life setting in which the data from a new subject are used only for testing the model. Empirical tests conducted in related research showed a large difference in the accuracies between epoch-level validation and subject-level validation [60].

In a study from Mehreen et al. [57], the authors also provide subject-level validation, and the accuracy achieved was 71.5% based on 15 frequency-domain features. The highest accuracy achieved in our research is shown in Table 9, and it was 65.45%, achieved by logistic regression. This 65.45% accuracy is relatively close to 71.5%, and it must be noted that it was obtained based only on the Index2 feature, with a simple algorithm and without any parameter optimization. Due to this, we are confident that the addition of our two multichannel ratio indices would lead to an improvement in all state-of-the-art drowsiness detection systems that use frequency-domain features. Again, our aim was not to create the best possible drowsiness detection model but to prove that the novel multichannel indices are better than the existing single-channel frequency-domain features.

The Equations (4) and (6) for these multichannel ratio indices, obtained after optimizing the parameters with the optimization algorithm, suggest that alpha and delta are two of the most important frequency power bands for drowsiness detection. Equation (6) suggests that delta power in the frontal region describes drowsiness better than in the central region, while alpha power in the occipital and central regions describes drowsiness better than in the frontal region.

These results are consistent with several previous research papers on drowsiness detection that reported the importance of increasing alpha power [22,35,61,62]. Delta power is usually only present in deep sleep stages [36], so some researchers studying drowsiness do not include delta in their research [63]. However, there is still much research that includes delta power. The increase in delta power is considered to be an indicator of drowsiness [4]. Our research found that theta and beta powers are not as good drowsiness indicators as alpha and delta powers, while many other research studies disagree. A decrease in beta power was found to be an indicator of drowsiness in [4,36,64,65] and an increase in theta power was found to be an indicator of drowsiness in [27,34,61,62,65]. Wang et al. [38], in their study of microsleep events, found that alpha and delta rhythms characterize microsleep events. As mentioned earlier, there is an inconsistency in terminology, and some researchers consider sleep stage S1 as drowsiness [5,6,7,8,9], while Johns [11] considers it equivalent to microsleep events in the driving scenario. We used the data from sleep stage S1 and referred to it in this research as drowsiness. Since our results suggest that delta and alpha are the most significant for the detection of drowsiness, as in the work of Wang et al. [38] on microsleep events, our work suggests that sleep stage S1 may be more similar to microsleep events than to drowsiness, but further research is needed to support this as a fact.

Apart from the indication that drowsiness is closely related to microsleep events, it may also be closely linked to driver fatigue. Some researchers even use the term fatigue as a synonym for drowsiness [66]. Fatigue is a consequence of prolonged physical or mental activity [67] and can lead to drowsiness [68]. Normally, rest and inactivity relieve fatigue, however, they exacerbate drowsiness [69]. Lal and Craig [70] found that delta and theta band activities increase significantly during fatigue. Craig et al. [71] reported significant changes in the alpha 1, alpha 2, theta and beta bands, while they did not find any significant changes in the delta band when observing driver fatigue. Simon et al. [68] report that alpha band power and alpha spindles correlate with fatigue.

These three research papers [68,70,71] all use visual inspection to define the ground truth of fatigue. This approach to defining the ground truth is prone to subjectivity. A similar problem occurs when drowsiness is defined by using subjective drowsiness ratings, such as the Karolinska sleepiness scale [72].

Driver drowsiness, driver fatigue and microsleep events are defined as different internal states of the brain, but show similar behavior when observing the features obtained from the EEG. Possible explanations could be that fatigue, drowsiness and microsleep have a similar effect on brain functions and cause the driver’s inability to function at the desired level. Most researchers of these three driver states only use frequency-domain features, while there are a number of other features (nonlinear features [30], spatiotemporal features [31] and entropies [32]) that could be used. Further studies with these features could find some features of the EEG signal that distinguish drowsiness, fatigue and microsleep. Distinguishing features of these three brain states could lead to the exact definitions of these terms. Precise and standardized definitions of fatigue, drowsiness and microsleep would help researchers to compare their work more easily.

**Figure 5:**
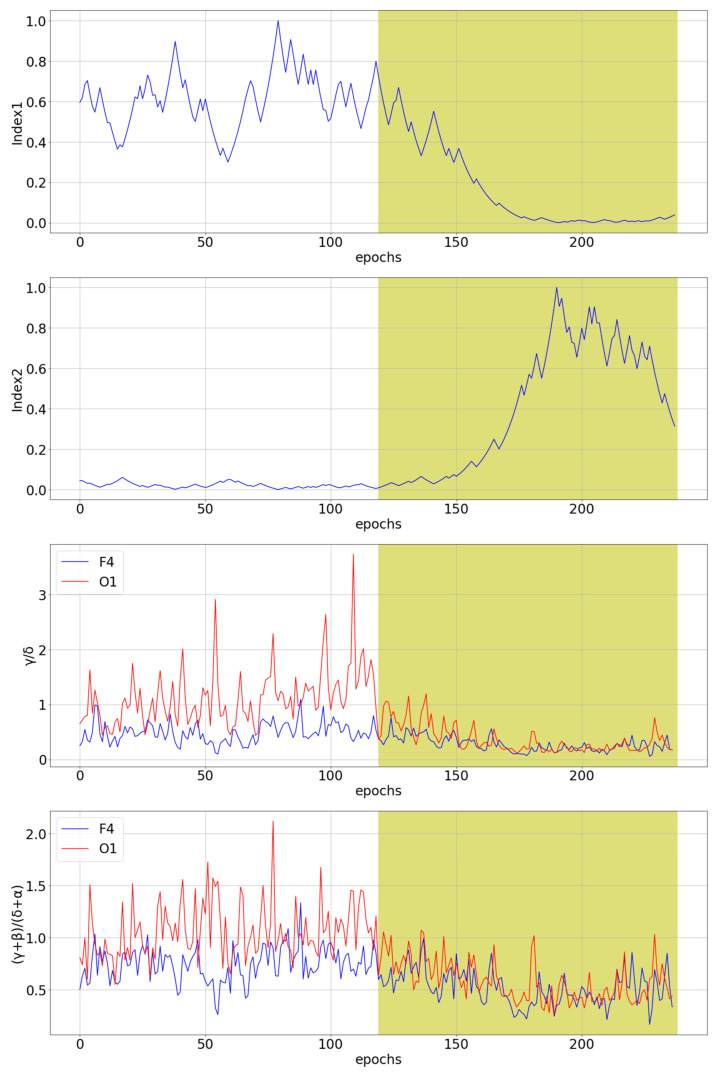
The comparison of the two novel multichannel indices with the best and the worst channel for γ/δ and (γ + β)/(δ + α) single-channel indices for subject tr04-0726. The white part of the diagram represents the awake state, while the yellow part of the diagram represents the stage 1 of sleep, i.e., the drowsiness state.

Figure 4 shows that the proposed procedure for creating the novel ratio indices has succeeded in creating step-like indices for a given subject. In addition to Index1 and Index2, which show desirable behavior, the indices (θ + α)/β and θ/β show similar, favorable behavior for a few channels. Figure 5 shows a comparison of novel ratio indices with the best and the worst channel for γ/δ and (γ + β)/(δ + α) single-channel indices for subject tr04-0726. Index1, index2, θ/α [36], (θ + α)/β, (θ + α)/(α + β) [37] and γ/δ [10] show similar behavior. These indices seem to detect drowsiness well, but with about a 50 epochs delay. Since several different single-channel indices that were previously shown to correlate with drowsiness together with two novel multichannel indices show the same delay in detecting drowsiness, this suggests that there may be shortcomings in the labeling of the initial signals. The manual for scoring sleep [42] provides guidelines for labeling, and it may be possible that the professionals who labeled the sleep signals labeled an approximate time of transition from the W state to the S1 state, as it is known that labeling any kind of several-hour-long EEG signal is a very tedious, hard and time-consuming job [73]. For this reason, the loose transition window is applied in the optimization algorithm, as described in Section 2.3.

The main shortcoming in applying our approach is the need to place six EEG electrodes on the driver’s scalp while driving. Apart from being intrusive, there is also a problem with noise in real-world applications that cannot be neutralized with the current state-of-the-art filter technology. All electrophysiological signals measured with wearable devices have a similar problem with intrusiveness and noise. ECG measurements, for example, are somewhat less susceptible to noise than EEG. Several recent works have shown that ECG can be used as a good predictor of sleep stages based on deep learning classifiers. Sun et al. [74] combined ECG with abdominal respiration and obtained a kappa value of 0.585, while Sridhar et al. [75] obtained a kappa value of 0.66. Combining EEG and ECG measurements has also been proposed in the context of driver drowsiness detection under simulator-based laboratory conditions [76]. Despite the problems of intrusiveness and noise susceptibility, research based on the electrophysiological signals brings a shift towards a precise definition of drowsiness. Once there is an exact definition of drowsiness or at least guidelines and manuals that accurately describe drowsiness (similar to the manuals for evaluating sleep stages), a big step will be taken to solve the problem of early detection of drowsiness [77]. It is doubtful that a wearable system based on electrophysiological signals will ever be widely used in real-world driving, but they still need to be developed. In our opinion, such wearable electrophysiological devices are more likely to be used for calibration/validation of non-intrusive systems (such as the driving performance-based or video-based systems) in controlled/simulated driving scenarios. In such scenarios, it is possible to control ambient noise, leading to a reduction in the effects of noise sensitivity.

An additional limitation of this work is that we were able to download data from 393 of 992 subjects completely, and only 28 of these 393 subjects were included in our study due to the inclusion condition that we described in Section 2.1 ”Dataset, Preprocessing and Feature Extraction”. Although it is a small subset of data, with the use of 12 subjects as a test set, we showed that the dataset is large enough to provide a good generalization (as seen in Table 3, Table 5 and Table 7). In a recent review paper about state-of-the-art drowsiness detection [33], the authors reviewed 39 papers, and the average number of subjects in the included works is 23.5, which also indicates that our number of subjects included in the current study (28) is acceptable.

## 5. Conclusions

This paper presented two novel multichannel ratio indices for the detection of drowsiness obtained by multi-objective optimization based on evolutionary computation. The results suggested that alpha and delta powers are good drowsiness indicators. The novel multichannel ratio indices were compared with seven existing single-channel ratio indices and showed better results in detecting drowsiness measured with precision and in the overall classification accuracy of both states using several machine learning algorithms. Our work suggests that a more precise definition of drowsiness is needed, and that accurate early detection of drowsiness should be based on multichannel frequency-domain ratio indices. The multichannel features also reduced the time needed for classification. The process of obtaining these indices by using a multi-objective optimization algorithm can also be applied to other areas of EEG signal analysis.

Research such as this, together with research on small hardware for physiology-based drowsiness detection, can eventually lead to an easy-to-use, non-intrusive device that reliably detects drowsiness. In addition, research on a reliable and standardized definition of drowsiness is needed and it would lead to improvements in the field of drowsiness detection.

## Figures and Tables

**Table 1:** The identification numbers of all the selected subjects. The training set is in the upper part and the test set is in the lower part of the table.

tr03-0092	tr03-0256	tr03-0876	tr03-1389
tr04-0649	tr04-0726	tr05-1434	Tr05-1675
tr07-0168	tr07-0458	tr07-0861	tr08-0021
tr08-0111	tr09-0175	tr10-0872	tr13-0204
tr04-0653	tr07-0127	tr09-0453	tr13-0170
tr05-0028	tr08-0157	tr12-0255	tr13-0508
tr05-0332	tr09-0328	tr12-0441	tr13-0653

**Table 2:** Statistical significance *p*-values were obtained by the Wilcoxon signed-rank test for distinguishing the awake state from the S1 state. The shaded green cells with bold text represent the lowest *p*-value for each subject in the training set. At the bottom, the index having *p*-values lower than α_p_ for most subjects is marked in the same way.

Subject	Index1	Index2	θ/α	β/α	(θ + α)/β	θ/β	(θ + α)/(α + β)	γ/δ	(γ + β)/(δ + α)
	This Work		Eoh et al. [36]		Jap et al. [37]			da Silveira et al. [10]	
tr03-0092	1.12 × 10^−6^	4.82 × 10^−3^	1.23 × 10^−4^	7.22 × 10^−1^	4.97 × 10^−2^	1.62 × 10^−08^	**1.41 × 10^−9^**	1.06 × 10^−6^	3.09 × 10^−4^
tr03-0256	3.92 × 10^−8^	**1.43 × 10^−13^**	2.42 × 10^−6^	6.88 × 10^−4^	1.11 × 10^−3^	4.04 × 10^−2^	3.60 × 10_−1_	9.08 × 10^−3^	1.51 × 10^−9^
tr03-0876	**1.37 × 10^−20^**	3.87 × 10^−17^	5.06 × 10^−3^	1.63 × 10^−7^	9.80 × 10^−14^	2.94 × 10^−6^	1.58 × 10^−5^	1.09 × 10^−1^	2.09 × 10^−1^
tr03-1389	**9.77 × 10^−11^**	1.35 × 10^−8^	9.64 × 10^−2^	2.06 × 10^−1^	2.86 × 10^−4^	1.27 × 10^−1^	1.91 × 10^−1^	1.82 × 10^−1^	3.01 × 10^−1^
tr04-0649	5.85 × 10^−21^	**4.11 × 10^−21^**	5.71 × 10^−12^	2.38 × 10^−9^	8.12 × 10^−7^	1.89 × 10^−1^	2.18 × 10^−5^	3.33 × 10^−7^	6.13 × 10^−3^
tr04-0726	2.96 × 10^−20^	3.19 × 10^−20^	5.90 × 10^−20^	2.31 × 10^−16^	9.40 × 10^−9^	2.78 × 10^−14^	2.62 × 10^−15^	2.36 × 10^−19^	**2.19 × 10^−20^**
tr05-1434	7.90 × 10^−10^	9.79 × 10^−13^	6.76 × 10^−9^	3.70 × 10^−10^	1.62 × 10^−1^	3.96 × 10^−17^	**2.00 × 10^−19^**	5.42 × 10^−17^	1.08 × 10^−11^
tr05-1675	1.71 × 10^−13^	1.85 × 10^−11^	1.24 × 10^−9^	7.82 × 10^−3^	1.48 × 10^−1^	1.10 × 10^−14^	**4.47 × 10^−16^**	1.58 × 10^−2^	4.62 × 10^−10^
tr07-0168	**2.88 × 10^−21^**	5.15 × 10^−21^	1.75 × 10^−13^	4.73 × 10^−11^	8.08 × 10^−6^	1.05 × 10^−8^	4.49 × 10^−11^	8.87 × 10^−16^	1.01 × 10^−1^
tr07-0458	8.34 × 10^−11^	**1.77 × 10^−16^**	1.66 × 10^−4^	1.77 × 10^−4^	5.51 × 10^−1^	1.32 × 10^−2^	6.62 × 10^−3^	3.68 × 10^−4^	4.17 × 10^−1^
tr07-0861	**2.88 × 10^−21^**	3.11 × 10^−21^	3.14 × 10^−3^	3.55 × 10^−7^	3.64 × 10^−2^	9.96 × 10^−8^	1.11 × 10^−6^	1.49 × 10^−17^	1.50 × 10^−12^
tr08-0021	**2.88 × 10^−21^**	**2.88 × 10^−21^**	4.09 × 10^−2^	2.54 × 10^−9^	4.55 × 10^−8^	3.34 × 10^−10^	4.91 × 10^−5^	4.19 × 10^−13^	2.10 × 10^−6^
tr08-0111	**2.88 × 10^−21^**	**2.88 × 10^−21^**	4.41 × 10^−2^	7.54 × 10^−5^	1.94 × 10^−20^	2.04 × 10^−20^	3.92 × 10^−4^	4.50 × 10^−15^	3.41 × 10^−3^
tr09-0175	7.78 × 10^−5^	7.92 × 10^−2^	4.64 × 10^−2^	3.10 × 10^−4^	**5.35 × 10^−14^**	2.23 × 10^−5^	2.68 × 10^−6^	7.18 × 10^−2^	1.30 × 10^−5^
tr × 10-0872	**2.62 × 10^−15^**	1.96 × 10^−14^	1.76 × 10^−2^	3.89 × 10^−2^	5.91 × 10^−3^	5.09 × 10^−6^	7.52 × 10^−5^	2.14 × 10^−5^	2.33 × 10^−5^
tr13-0204	1.71 × 10^−3^	6.62 × 10^−1^	6.30 × 10^−4^	4.91 × 10^−5^	6.36 × 10^−5^	2.91 × 10^−10^	**2.63 × 10^−10^**	5.59 × 10^−1^	2.16 × 10^−2^
No. subjects with *p* < 6.9 × 10^−5^	**14**	13	6	9	8	12	11	9	8

**Table 3:** Statistical significance *p*-values were obtained by the Wilcoxon signed-rank test for distinguishing the awake state from the S1 state. The shaded green cells with bold text represent the lowest *p*-value for each subject in the test set. At the bottom, the index having *p*-values lower than α_p_ for most subjects is marked in the same way.

Subject	Index1	Index2	θ/α	β/α	(θ + α)/β	θ/β	(θ + α)/(α + β)	γ/δ	(γ + β)/(δ + α)
	This Work		Eoh et al. [36]		Jap et al. [37]			da Silveira et al. [10]	
tr04-0653	3.19 × 10^−9^	2.71 × 10^−9^	2.61 × 10^−7^	1.80 × 10^−5^	6.83 × 10^−1^	3.63 × 10^−9^	2.17 × 10^−8^	2.69 × 10^−6^	**3.33 × 10^−10^**
tr05-0028	**1.26 × 10^−9^**	2.36 × 10^−7^	3.78 × 10^−3^	2.99 × 10^−1^	3.45 × 10^−1^	3.56 × 10^−1^	5.16 × 10^−2^	1.70 × 10^−1^	4.36 × 10^−2^
tr05-0332	**2.88 × 10^−21^**	**2.88 × 10^−21^**	8.04 × 10^−16^	2.10 × 10^−5^	2.66 × 10^−3^	7.39 × 10^−14^	8.17 × 10^−18^	1.84 × 10^−16^	3.00 × 10^−16^
tr07-0127	**3.71 × 10^−18^**	1.27 × 10^−15^	8.92 × 10^−2^	1.44 × 10^−2^	3.44 × 10^−5^	1.38 × 10^−2^	2.51 × 10^−5^	9.07 × 10^−16^	3.36 × 10^−16^
tr08-0157	**2.88 × 10^−21^**	**2.88 × 10^−21^**	6.51 × 10^−5^	1.26 × 10^−7^	9.66 × 10^−1^	6.67 × 10^−3^	1.85 × 10^−3^	2.82 × 10^−11^	5.92 × 10^−11^
tr09-0328	**1.80 × 10^−10^**	1.14 × 10^−2^	7.90 × 10^−10^	9.56 × 10^−7^	1.70 × 10^−7^	2.01 × 10^−1^	1.92 × 10^−3^	3.54 × 10^−2^	7.01 × 10^−5^
tr09-0453	2.73 × 10^−1^	7.80 × 10^−4^	1.63 × 10^−1^	2.96 × 10^−1^	1.03 × 10^−7^	3.89 × 10^−2^	3.17 × 10^−2^	**8.30 × 10^−16^**	6.45 × 10^−9^
tr12-0255	1.37 × 10^−2^	5.55 × 10^−10^	4.31 × 10^−19^	2.17 × 10^−18^	1.89 × 10^−16^	**2.20 × 10^−19^**	8.22 × 10^−19^	1.11 × 10^−7^	2.33 × 10^−7^
tr12-0441	2.76 × 10^−11^	9.26 × 10^−9^	6.95 × 10^−4^	6.89 × 10^−1^	2.46 × 10^−3^	**3.63 × 10^−13^**	1.53 × 10^−4^	2.13 × 10^−3^	5.68 × 10^−8^
tr13-0170	7.59 × 10^−7^	3.60 × 10^−5^	3.74 × 10^−2^	4.73 × 10^−2^	1.91 × 10^−4^	9.71 × 10^−4^	2.16 × 10^−2^	**6.07 × 10^−17^**	5.23 × 10^−16^
tr13-0508	2.69 × 10^−1^	7.61 × 10^−2^	1.87 × 10^−5^	1.99 × 10^−2^	**1.10 × 10^−9^**	9.17 × 10^−2^	6.22 × 10^−5^	7.26 × 10^−5^	7.26 × 10^−2^
tr13-0653	1.09 × 10^−16^	**7.36 × 10^−20^**	4.90 × 10^−8^	4.16 × 10^−4^	8.05 × 10^−2^	1.66 × 10^−2^	1.03 × 10^−9^	3.47 × 10^−2^	1.40 × 10^−5^
No. subjects with *p* < 6.9 × 10^−5^	**9**	**9**	7	5	5	4	6	7	**9**

**Table 4:** The classification accuracy was obtained with the XGBoost algorithm for each subject in the training set. The shaded green cells with bold text show the highest accuracy obtained for each subject. At the bottom, the best mean accuracy for each ratio index is marked in the same way.

Subject	Index1	Index2	θ/α	β/α	(θ + α)/β	θ/β	(θ + α)/(α + β)	γ/δ	(γ + β)/(δ + α)
	This Work		Eoh et al. [36]		Jap et al. [37]			da Silveira et al. [10]	
tr03-0092	0.5420	0.5252	**0.6387**	0.5168	0.5504	0.6092	0.6050	0.4684	0.5527
tr03-0256	0.5924	0.6303	0.5840	0.5504	0.5672	**0.6345**	**0.6345**	0.4473	0.4346
tr03-0876	0.6387	0.5672	0.6008	0.6218	0.4748	**0.6471**	0.6303	0.5781	0.5992
tr03-1389	0.3487	0.3908	0.4874	0.5588	0.5042	0.5378	0.4664	**0.5696**	0.5148
tr04-0649	0.6975	**0.8025**	0.5462	0.5462	0.5630	0.4832	0.5210	0.6118	0.5527
tr04-0726	**0.7983**	0.7605	0.6681	0.6176	0.5966	0.6134	0.6765	0.7637	0.7511
tr05-1434	0.3739	0.3697	0.4160	0.6218	**0.7059**	0.5882	0.6933	0.5485	0.5063
tr05-1675	0.6849	0.6513	0.6933	0.5546	0.6218	0.5630	**0.7227**	0.5781	0.5781
tr07-0168	0.7773	**0.8193**	0.6008	0.5504	0.5630	0.6092	0.6303	0.4473	0.5105
tr07-0458	0.3109	0.2689	**0.5378**	0.4748	0.5084	**0.5378**	0.5504	0.4684	0.4979
tr07-0861	0.6933	**0.7269**	0.5378	0.5420	0.5504	0.5168	0.5714	0.6540	0.6160
tr08-0021	**0.7857**	0.6387	0.5630	0.4874	0.4664	0.3866	0.4748	0.6329	0.6245
tr08-0111	0.6891	**0.8025**	0.7143	0.7101	0.7227	0.5462	0.4748	0.6287	0.6118
tr09-0175	0.6050	0.5252	0.5966	0.4748	0.5420	0.6218	0.6008	0.5401	**0.6498**
tr10-0872	**0.6134**	0.6008	0.5084	0.5168	0.4874	0.5252	0.4832	0.5274	0.4810
tr13-0204	0.5042	0.4538	**0.6597**	0.4790	0.6387	0.6471	0.6303	0.5570	0.5570
Average	**0.6035**	0.5959	0.5846	0.5515	0.5664	0.5667	0.5853	0.5638	0.5649

**Table 5:** The classification accuracy was obtained with the XGBoost algorithm for each subject in the test set. The shaded green cells with bold text show the highest accuracy obtained for each subject. At the bottom, the best mean accuracy for each ratio index is marked in the same way.

Subject	Index1	Index2	θ/α	β/α	(θ + α)/β	θ/β	(θ + α)/(α + β)	γ/δ	(γ + β)/(δ + α)
	This Work		Eoh et al. [36]		Jap et al. [37]			da Silveira et al. [10]	
tr04-0653	0.5672	**0.6345**	0.6303	0.5042	0.5168	0.5840	0.5630	0.5612	0.5738
tr05-0028	0.4454	0.4202	0.5294	**0.5588**	0.4664	0.4076	0.5462	0.5021	0.5443
tr05-0332	0.8067	**0.8277**	0.7563	0.5630	0.5294	0.6555	0.6134	0.5654	0.6118
tr07-0127	0.5462	**0.6092**	0.4916	0.5294	0.5252	0.5000	0.4118	0.4304	0.4051
tr08-0157	0.6303	**0.6681**	0.5294	0.5294	0.5000	0.5084	0.5294	0.5232	0.4810
tr09-0328	**0.6050**	0.5084	0.5966	0.5168	0.5000	0.5042	0.5672	0.5738	0.5401
tr09-0453	0.5588	0.5252	0.5714	0.5420	0.5294	0.5504	**0.5840**	0.5105	0.4557
tr12-0255	0.5420	0.5546	**0.6639**	0.5462	0.4748	0.5630	0.5924	0.6329	0.5654
tr12-0441	**0.6891**	0.5756	0.5840	0.5168	0.5378	0.5630	0.5798	0.6498	0.5274
tr13-0170	0.6008	0.5630	0.5924	0.5546	0.6261	**0.6471**	0.5336	0.5612	0.4430
tr13-0508	0.4538	0.5084	**0.6555**	0.6008	0.5588	0.6261	0.6092	0.5654	0.5443
tr13-0653	**0.6807**	0.6303	0.5210	0.5462	0.5630	0.5336	0.6008	0.5359	0.5063
Average	**0.5938**	0.5854	0.5935	0.5424	0.5273	0.5536	0.5609	0.5510	0.5165

**Table 6:** The precision of drowsiness detection was obtained with the XGBoost algorithm for each subject in the training set. The shaded green cells with bold text show the highest precision obtained for each subject. At the bottom, the best mean precision for each ratio index is marked in the same way.

Subject	Index1	Index2	θ/α	β/α	(θ + α)/β	θ/β	(θ + α)/(α + β)	γ/δ	(γ + β)/(δ + α)
	This Work		Eoh et al. [36]		Jap et al. [37]			da Silveira et al. [10]	
tr03-0092	0.5439	0.5439	0.5439	0.5439	0.5439	0.5439	0.5439	0.5439	0.5439
tr03-0256	0.6222	**0.6348**	0.5676	0.5349	0.5571	0.6127	0.6096	0.4270	0.3974
tr03-0876	0.6514	0.5800	0.6765	0.5973	0.4808	**0.7397**	0.7123	0.5804	0.6264
tr03-1389	0.2273	0.3774	0.4906	0.5455	0.5034	0.5391	0.4556	**0.5519**	0.5120
tr04-0649	0.7582	0.8273	0.5733	0.7895	0.7778	0.4878	0.5294	**0.9063**	0.6000
tr04-0726	0.8318	**1.0000**	0.7128	0.6373	0.6055	0.6627	0.7333	0.8370	0.7706
tr05-1434	0.4051	0.4083	0.1429	0.6028	0.7168	0.7692	**0.7805**	0.6571	0.5027
tr05-1675	0.6642	0.6011	0.6885	0.5607	0.6559	0.5862	**0.7265**	0.5425	0.5433
tr07-0168	0.7500	**0.7923**	0.5759	0.5375	0.5397	0.5730	0.5963	0.4488	0.5156
tr07-0458	0.2816	0.0492	0.5437	0.4789	0.5088	0.5446	**0.5732**	0.4500	0.4930
tr07-0861	0.6264	**0.6688**	0.5338	0.5342	0.5349	0.5105	0.5521	0.6216	0.5780
tr08-0021	**0.7464**	0.6854	0.6119	0.4717	0.4535	0.4000	0.4688	0.6325	0.6355
tr08-0111	0.6692	**0.8214**	0.7297	0.8205	0.7912	0.5314	0.4840	0.6500	0.5985
tr09-0175	**0.6404**	0.5263	0.5742	0.4762	0.5379	0.6142	0.6053	0.5273	0.6207
tr10-0872	**0.6174**	0.5909	0.5045	0.5130	0.4892	0.5254	0.4882	0.5349	0.4627
tr13-0204	0.5054	0.4545	0.6357	0.4820	0.6170	**0.6636**	0.6348	0.6032	0.5970
Average	0.5963	**0.5976**	0.5691	0.5704	0.5821	0.5815	0.5934	0.5946	0.5623

**Table 7:** The precision of drowsiness detection was obtained with the XGBoost algorithm for each subject in the test set. The shaded green cells with bold text show the highest precision obtained for each subject. At the bottom, the best mean precision for each ratio index is marked in the same way.

Subject	Index1	Index2	θ/α	β/α	(θ + α)/β	θ/β	(θ + α)/(α + β)	γ/δ	(γ + β)/(δ + α)
	This Work		Eoh et al. [36]		Jap et al. [37]			da Silveira et al. [10]	
tr04-0653	0.5769	0.6569	**0.6742**	0.5030	0.5133	0.6000	0.5862	0.5795	0.5914
tr05-0028	0.3818	0.4296	0.5178	0.5385	0.4762	0.4214	0.5364	0.5000	**0.5862**
tr05-0332	0.7483	**0.9333**	0.7905	0.5547	0.5321	0.7846	0.6709	0.8571	0.8824
tr07-0127	0.5401	**0.6512**	0.4722	0.5294	0.5231	0.5000	0.3600	0.4309	0.4207
tr08-0157	0.5812	**0.6087**	0.5158	0.5574	0.5000	0.5048	0.5153	0.5122	0.4886
tr09-0328	**0.6147**	0.5078	0.5650	0.5137	0.5000	0.5041	0.5678	0.5620	0.5372
tr09-0453	0.5398	0.5176	0.5436	0.5301	0.5248	0.5306	**0.5538**	0.5049	0.4721
tr12-0255	0.5316	0.5351	**0.6054**	0.5342	0.4826	0.5393	0.5545	0.5886	0.5389
tr12-0441	**0.8000**	0.5789	0.5633	0.5093	0.5249	0.5478	0.5785	0.6496	0.5231
tr13-0170	0.6333	0.5466	0.5724	0.5355	0.6056	0.6296	0.5313	**0.6944**	0.3478
tr13-0508	0.4337	0.5091	**0.6331**	0.5674	0.5443	0.6154	0.6140	0.5478	0.5352
tr13-0653	**0.7048**	0.6000	0.5177	0.5314	0.5419	0.5213	0.5845	0.5392	0.5037
Average	**0.5905**	0.5896	0.5809	0.5337	0.5224	0.5582	0.5544	0.5805	0.5356

**Table 8:** The average time of 100 executions of the XGBoost classifier’s initialization, training, classifications on the test subject and calculation of classification accuracy, expressed in milliseconds. The shaded green cells with bold text represent the best values for each subject and the best average value.

Subject	Index1	Index2	θ/α	β/α	(θ + α)/β	θ/β	(θ + α)/(α + β)	γ/δ	(γ + β)/(δ + α)
	This Work		Eoh et al. [36]		Jap et al. [37]			da Silveira et al. [10]	
tr03-0092	**86.3772**	86.9689	122.7764	124.0313	129.9221	129.5543	130.6956	128.6490	128.9000
tr03-0256	**86.7446**	87.1034	123.3161	123.2911	130.3844	130.2867	130.6147	128.6415	128.7518
tr03-0876	**86.3508**	87.0188	122.6970	123.5249	130.2485	130.7789	130.6456	128.7160	128.3419
tr03-1389	**85.9344**	86.8811	122.1586	123.8243	130.4414	130.1170	131.3382	129.2281	128.8390
tr04-0649	**86.9833**	87.5527	123.6650	124.0832	130.2565	130.0574	130.2316	129.2234	128.8357
tr04-0726	**86.5498**	87.5921	123.7690	123.6945	129.6285	129.6750	130.6256	128.9002	128.6363
tr05-1434	**86.5450**	86.6549	123.0505	130.6853	131.9267	130.6205	131.3138	130.6215	131.3438
tr05-1675	**87.0534**	87.4627	123.3135	130.0399	130.4660	129.1552	129.5943	130.5365	128.9256
tr07-0168	**86.9143**	87.2251	122.9559	129.6185	130.5158	130.1070	129.7780	129.6381	128.7795
tr07-0458	**86.5651**	86.8690	122.6074	129.9533	130.1468	130.2667	130.4915	128.8906	129.5788
tr07-0861	**86.8634**	87.2801	122.7545	130.2319	130.0104	128.1135	129.9124	129.3949	128.7760
tr08-0021	**86.9239**	88.9566	123.0910	130.0868	129.1948	130.0221	130.1670	129.3241	129.1652
tr08-0111	**86.6697**	87.4626	122.7216	130.3879	130.5019	130.4011	129.8419	128.8413	129.1940
tr09-0175	**87.3240**	87.3827	123.4803	129.6729	130.9953	130.1271	131.1785	128.7062	128.9786
tr10-0872	**86.9690**	87.5381	124.0918	130.5091	129.4638	130.6490	130.2964	129.4843	129.3599
tr13-0204	**87.2509**	**87.1135**	123.2010	131.7928	130.4062	131.4087	130.3568	128.9199	128.1530
Average	**86.7512**	87.3164	123.1031	127.8392	130.2818	130.0838	130.4426	129.2322	129.0350

**Table 9:** The average accuracy was obtained on the test set with different classification algorithms. Each row is colored with a pallet of colors ranging from dark green for the highest number in the row to dark red for the lowest number in the row. The algorithms are: NB—Naïve Bayes, KNN—k nearest neighbors, Logistic—logistic regression, DT—decision tree, RF—random forest and SVM—support vector machine.

Algorithm	Index1	Index2	θ/α	β/α	(θ + α)/β	θ/β	(θ + α)/(α + β)	γ/δ	(γ + β)/(δ + α)
	This Work		Eoh et al. [36]		Jap et al. [37]			da Silveira et al. [10]	
NB	0.6399	0.6535	0.5947	0.5462	0.5432	0.5308	0.5663	0.5316	0.5277
KNN	0.5785	0.5840	0.5588	0.5387	0.5399	0.5452	0.5525	0.5378	0.5277
Logistic	0.6396	0.6543	0.6029	0.5131	0.5383	0.5626	0.5735	0.5793	0.5613
DT	0.5717	0.5629	0.5456	0.5050	0.5074	0.5420	0.5267	0.5356	0.5223
RF	0.5719	0.5659	0.5762	0.5380	0.5360	0.5549	0.5501	0.5321	0.5222
SVM	0.6325	0.6526	0.6200	0.5695	0.5714	0.5731	0.5801	0.5541	0.5478

## Data Availability

The data used in this paper were obtained from the PhysioNet portal [47], from the 2018 PhysioNet computing in cardiology challenge [48], at: https://physionet.org/content/challenge-2018/1.0.0/ (accessed on 24 September 2021).
